# Strong development of research based on national quality registries in Sweden

**DOI:** 10.1080/03009734.2018.1520761

**Published:** 2018-10-01

**Authors:** Jack Lysholm, Bertil Lindahl

**Affiliations:** a Department of Public Health and Clinical Medicine, Umeå University, Umeå, Sweden;; b Department of Medical Sciences and Uppsala Clinical Research Center, Uppsala University, Uppsala, Sweden

**Keywords:** Quality registries; research; peer-reviewed journals

## Abstract

The aim of the present paper is to describe how the use of national quality registries (NQRs) for research has evolved over the past decade in Sweden. All Swedish NQRs have reported their scientific activity (publications per year in peer-reviewed scientific journals) to the Swedish Association of Local Authorities and Regions since 2009, and the present report is based on available data from 2009–2016. The yearly number of publications of the 69 registries active in 2009 has increased from 121 to 496 in 2016. Seventeen of these registries published more than 10 papers in 2016; however, 12 NQRs did not publish any papers in 2016. An additional 77 papers were published in 2016 by the 34 NQRs started after 2009. In summary, there has been a strong development of quality registry-based research in Sweden over the last decade. However, there is still room for further increase of the use of research based on NQRs in Sweden.

According to Swedish law a quality registry is a structured collection of information with the primary aim to develop and safeguard the quality of care. However, once the information has been gathered, data in the registry can also be used for statistics and research. Quality registries that have applied for and been granted national funding are referred to as national quality registries (NQRs). Since the first NQR in Sweden, the Knee Arthroplasty Registry, was started in 1975 many NQRs have been initiated, covering many different areas of the Swedish health care sector. Currently (2018), there are 108 ongoing NQRs in Sweden.

The participation in a NQR is voluntary both for health care providers and patients, which differentiates them from health and population registries managed by government agencies like the National Board of Health and Welfare (e.g. Cause of Death registry and National Patient registry) and Statistics Sweden (e.g. on socioeconomic condition). Participation in the last-mentioned registries is mandatory by law both for the health care providers and for patients. In contrast, for NQR an “opt-out” principle is used; it is assumed that the patient gives consent unless he/she does not actively say no to participation after being informed of registration in a NQR.

A high-quality NQR contains detailed data on the individual patient that can be very useful for observational studies, especially if combined with data from the mandatory health registries in Sweden. Recently, a few NQRs have also been used for performing pragmatic randomized clinical trials incorporated in the registry (1).

For a long time NQRs were underutilized for research. However, in the last decade there have been several efforts to increase the use of NQRs for research. Therefore, the aim of the present paper was to describe how the use of NQRs for research has evolved over the past decade.

## Method

Swedish NQRs have reported their scientific activity (number of publications per year) to the Swedish Association of Local Authorities and Regions (SALAR) since 2009. As the use of a NQR for research has an effect on both the level of certification (a criterion-based classification system, which certifies the register as level 1–3, where 1 is the highest level) of the register and the annual grant from SALAR and the Swedish government, it is important for the register to compile and report their list of scientific publications for the previous year. There were 69 registers in 2009 that still reported in 2016. These lists of publications, available via SALAR, were scrutinized in order to exclude all publications that did not qualify by being included in peer-reviewed scientific journals. Titles and if necessary abstracts were checked to ensure that the paper to a significant degree was based on data from a NQR. Moreover, the list was cleared from double registrations (e.g. e-publication ahead of print). To obtain the total number of publications from Swedish NQRs in 2016, peer-reviewed scientific publications reported by the 34 NQRs started since 2009 were added.

## Results

**Figure 1.**
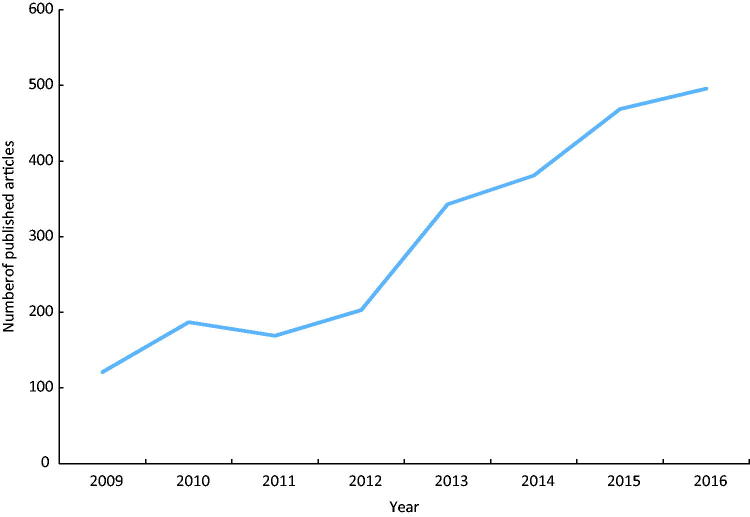
Yearly numbers of peer-reviewed publications based on Swedish national quality registers (*n* = 69) 2009–2016.

**Figure 2.**
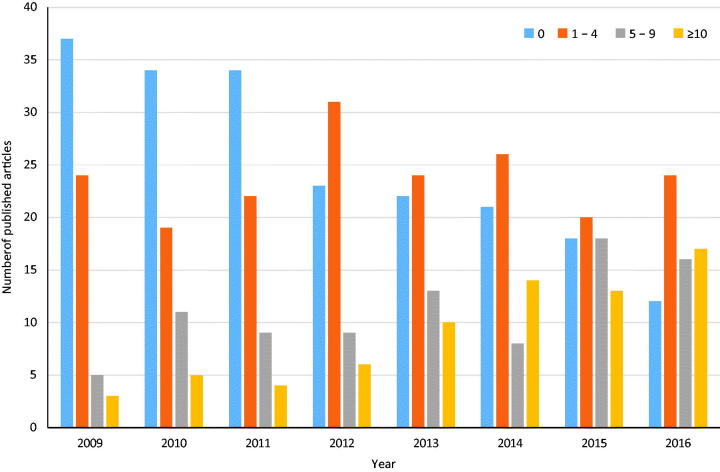
The Swedish national quality registers were divided into four subgroups characterized by the yearly number of published peer-reviewed articles. Blue bars denote no articles, brown bars 1–4 articles, grey bars 5–9 articles, and yellow bars 10 or more articles.

The yearly number of publications in peer-reviewed journals based on results from NQRs increased from 121 in 2009 to 496 in 2016 (Figure 1). There was a special national campaign in order to strengthen NQRs in 2012–2016. During this campaign the number of publications increased almost three-fold (by a factor of 2.93). Data from many NQRs were extensively used for research. The number of NQRs reporting 10 or more articles increased from 3 in 2009 to 17 in 2016. Moreover, during the same time period the number of NQRs reporting no publications at all decreased from 37 to 12 (Figure 2).

At the end of 2016 another 34 NQRs had been included during the campaign period. Some of them had been started recently, and still had a lot of development to do in terms of participation, coverage, and data quality. In 2016 data from these NQRs were reported in 77 articles. Twenty-two of these 34 registers reported no publications at all, but a few (*n* = 3) reported 10 or more publications. The total number of peer-reviewed papers based on data from all ongoing NQRs in 2016 was 573.

## Discussion

There has been an impressive increase in the number of peer-reviewed publications from the Swedish NQRs during the study period. The increase is due to the fact that the proportion of registers reporting publications, as well as the number of publications per register, has increased. Unfortunately, the increased publication activity does not guarantee a corresponding increase in the quality of research. However, many papers from the NQRs were published in high-ranked journals like *New England Journal of Medicine*, *JAMA*, and *The Lancet* (2–6). Furthermore, a review of the Swedish NQRs performed by the Swedish Agency for Health and Care Services Analysis stated that the papers based on NQRs were published in journals with higher impact factors than the average clinical research paper in Sweden (7). Despite this strong development, there is still room for a further increase in the use of NQR for research, since many NQRs still publish few or even no papers.

There are several possible explanations for this favorable development of the quality registry-based research. *Firstly*, during this time period a special national campaign in order to strengthen NQRs (2012–2016) took place, in which the funding of the NQRs was substantially increased. Although this campaign was primarily focused on strengthening the NQR for their use in improving the quality of health care, the resulting increase in e.g. data quality and coverage has also been beneficial for research. In addition, several efforts were also aimed directly at strengthening research, e.g. an annual research meeting and increased support to the regional registry competence centers (e.g. Uppsala Clinical Research Center) for statistical competence and capacity. *Secondly*, simultaneously, an increased interest among funding agencies to fund registry-based research has been noticed. *Thirdly*, and most importantly, more NQRs have achieved the maturity required to enable high-quality research to be conducted. There is a substantial lag between the start of a registry and the point when it has reached high coverage and enough high-quality data have been gathered.

In summary, during the last decade there has been a strong development of the quality registry-based research in Sweden. However, there is still room for further increase of the use of research based on NQRs in Sweden.
